# Infection by Brazilian and Dutch swine hepatitis E virus strains induces haematological changes in *Macaca fascicularis*

**DOI:** 10.1186/1471-2334-13-495

**Published:** 2013-10-23

**Authors:** Lilian G de Carvalho, Renato S Marchevsky, Debora RL dos Santos, Jaqueline M de Oliveira, Vanessa S de Paula, Leilane M Lopes, Wilhelmus HM Van der Poel, Jorge E González, Maria S Munné, Julio Moran, Ana Carolina R A Cajaraville, Marcelo Pelajo-Machado, Oswaldo G Cruz, Marcelo A Pinto

**Affiliations:** 1 Centre for Laboratory Animal Breeding, Department of Primatology, Oswaldo Cruz Foundation, Rio de Janeiro, Brazil; 2 Laboratory of Neurovirulence, Institute of Technology on Immunobiologicals, Bio-Manguinhos, Oswaldo Cruz Foundation, Rio de Janeiro, Brazil; 3 Laboratory of Veterinary Viruses, Department of Veterinary Microbiology and Immunology, UFRRJ, Rio de Janeiro, Brazil; 4 Laboratory of Technological Development in Virology, Oswaldo Cruz Institute/Oswaldo Cruz Foundation, Rio de Janeiro, Brazil; 5 Central Veterinary, Institute of Wageningen University and Research Centre, Wageningen, The Netherlands; 6 National Reference Laboratory in Viral Hepatitis, National Institute of Infectious Diseases, Buenos Aires, Argentina; 7 Dr. Julio Moran Laboratories, Ebmatingen, Zurich, Switzerland; 8 Laboratory of Virological Technology, Institute of Technology on Immunobiologicals, Bio-Manguinhos, Oswaldo Cruz Foundation, Rio de Janeiro, Brazil; 9 Laboratory of Pathology, Oswaldo Cruz Institute/Oswaldo Cruz Foundation, Rio de Janeiro, Brazil; 10 Programme of Scientific Computation, Oswaldo Cruz Foundation, Rio de Janeiro, Brazil

**Keywords:** Swine HEV, Human HEV, Lymphopenia, Fulminant hepatic failure, Genotype 3, *Macaca fascicularis*

## Abstract

**Background:**

Hepatitis E virus (HEV) has been described as an emerging pathogen in Brazil and seems to be widely disseminated among swine herds. An autochthonous human case of acute hepatitis E was recently reported. To obtain a better understanding of the phenotypic profiles of both human and swine HEV strains, a experimental study was conducted using the animal model, *Macaca fascicularis*.

**Methods:**

Six cynomolgus monkeys (*Macaca fascicularis*) were inoculated intravenously with swine HEV genotype 3 that was isolated from naturally and experimentally infected pigs in Brazil and the Netherlands. Two other monkeys were inoculated with HEV genotype 3 that was recovered from Brazilian and Argentinean patients with locally acquired acute and fulminant hepatitis E. The haematological, biochemical, and virological parameters of all animals were monitored for 67 days.

**Results:**

Subclinical hepatitis was observed in all monkeys after inoculation with HEV genotype 3 that was recovered from the infected swine and human patients. HEV RNA was detected in the serum and/or faeces of 6 out of the 8 cynomolgus monkeys between 5 and 53 days after inoculation. The mild inflammation of liver tissues and elevations of discrete liver enzymes were observed. Seroconversions to anti-HEV IgM and/or IgG were detected in 7 animals. Reactivities to anti-HEV IgA were also detected in the salivary samples of 3 animals. Interestingly, all of the infected monkeys showed severe lymphopenia and a trend toward monocytosis, which coincided with elevations in alanine aminotransferase and antibody titres.

**Conclusions:**

The ability of HEV to cross the species barrier was confirmed for both the swine (Brazilian and Dutch) and human (Argentinean) strains, thus reinforcing the zoonotic risk of hepatitis E in South America. Cynomolgus monkeys that were infected with HEV genotype 3 developed subclinical hepatitis that was associated with haematological changes. Haematological approaches should be considered in future studies of HEV infection.

## Background

Infection with hepatitis E virus (HEV) has been well documented in endemic areas, such as southeastern and central Asia, the Middle East, the northern and western parts of Africa, and North America [1]. In areas of poor sanitation, epidemics occur periodically, and infection is mainly transmitted via the faecal contamination of water. However, the global burden of HEV infection is more influenced by sporadically transmitted hepatitis cases than by epidemics [2]. In South America and other non-endemic areas, epidemics of hepatitis E have not yet been reported. Nevertheless, sporadic cases of both acute, self-limited hepatitis and fulminant hepatic failure have been reported in Venezuela, Brazil, and Argentina [3]. In most of these cases, the human isolates were related to swine genotype 3 HEV strains from the same region.

In Brazil, HEV genotype 3 seems to be widely disseminated. The virus has been detected in swine and effluent samples from farms and slaughterhouses. Among swine handlers, the anti-HEV IgG seroprevalence is estimated to be 6 to 8% [4,5] and is approximately 2% in urban areas [6,7]. Although national data show high numbers of alleged cases, the true incidence of hepatitis E in the Brazilian population is uncertain because a testing protocol for HEV has not yet been established. A retrospective study of sera from patients with non-A-C acute hepatitis from 2005–2009 led to the identification of the first autochthonous human case of acute HEV infection. The genetic relatedness of this strain and the genotype 3 HEV in pigs is indicative of the zoonotic circulation of HEV in Brazil [8-10].

Infection studies using *Macaca fascicularis*, *Aotus trivirgatus*, *Macaca mulatta*, and *Pan troglodytes* that have evaluated the course of HEV infection have been previously described [11-16]. These studies have created an experimental foundation for the understanding of the immunological behaviour of HEV, including the cross-reactivities among different isolated strains [17,18] and the conferral of cross-immunity among genotypes 3 and 4 in swine hosts [19]. HEV genotype 3 shows dose-dependent infectivity [20] and is able to cross species barriers [21-23], causing infection in species such as the cynomolgus monkey [24], which is considered to be a well-established model for human HEV infection.

Currently, most experimental studies of HEV investigate only virological and hepatic parameters. However, there is limited information available describing the extra-hepatic manifestations of HEV infection, including a few reports highlighting other clinical findings, such as pancreatitis, thrombocytopenia, aplastic anaemia, acute thyroiditis, glomerulonephritis, and neurologic disorders, as reviewed by Dalton et al. [25]. To obtain a better understanding of the phenotypic profiles of both human and swine Brazilian HEV strains, a descriptive study was conducted in the experimental model *Macaca fascicularis*. Human and swine HEV samples from Argentina and the Netherlands were also used as inocula. This article describes, for the first time, haematological disorders that could be interpreted as extra-hepatic manifestations of HEV infection.

## Methods

### Animals

Ten clinically healthy young adult cynomolgus monkeys, each weighing 1.5–6.0 kg, were provided for use in this study by the Non-Human Primates Breeding Service Centre (CECAL) of the Oswaldo Cruz Foundation (Fiocruz), Rio de Janeiro, Brazil. The study protocol was approved (L-0033/07) by the Ethics Committee for Animal Use (CEUA), Fiocruz, and was conducted in strict accordance with the recommendations from the Guide for the Care and Use of Laboratory Animals of the Brazilian Society of Science in Laboratory Animals (SBCAL) and the National Council for Control of Animal Experimentation (CONCEA, Brazil). The animals that were selected for the study were free of simian immunodeficiency virus (SIV) and simian type D retrovirus (SRV/D). They were also negative for anti-HEV IgG in their sera and had no inflammatory changes in their pre-study liver biopsies. During the study and quarantine periods, the monkeys were maintained at Animal Biosafety Level 2 in CECAL, Fiocruz, and were kept individually in stainless steel squeeze-back cages in a climate-controlled room (temperature of 22 ± 1°C and humidity 55 ± 5%) with a 12 h light/dark cycle.

### Inocula

The Brazilian swine HEV (Br-swine) genotype 3b (GenBank EF591853.1) strain was isolated from a faecal suspension that was obtained from a naturally infected pig from a commercial farm in Rio de Janeiro state [12]. The Dutch swine HEV (D-swine) genotype 3 (GenBank DQ996399) strain, which was kindly supplied by the Central Veterinary Institute of Wageningen University and Research Centre, the Netherlands, was obtained from an experimentally infected pig [23].

The Brazilian human HEV (Br-human) genotype 3b (GenBank GQ421465) strain was isolated from a 1-ml serum sample that was obtained from a 30-year-old male with acute hepatitis E [26]. The Argentinean human HEV (Ar-human) sample was kindly provided by the Dr. Carlos Malbran Institute, Buenos Aires, and was prepared from a pool of 1 ml of serum and the faeces of a 3-month-old female with fulminant acute hepatic failure (Table 1). This study was approved by the institutional review boards (CEP-Fiocruz No. 22/03), and a signed informed consent form was obtained from each participant.

**Table 1:** Sources of hepatitis E virus inocula used to infect the cynomolgus monkeys

**Source**	**Inocula**	**Anti-HEV serology**	**HEV RT-PCR/real-time PCR**	**Genotype**	**Age of monkey (years)**	**Animal ID**
Swine	Pooled faeces (3.0 ml)	_	HEV RNA+/10^5^ copies/ml	3	15	I3
					18	Q11
					2	X15
Swine	Pooled faeces (3.0 ml)	_	HEV RNA+/10^6^ copies/ml	3	11	O1
					17	G3
					19	F3
Child, female, 3 months old (FALF^*^)	Serum and faeces (1.0 ml)	Undetectable	Undetectable/10^3^ copies/ml	3	7	R7
Adult, male, 30 years old (AH^**^)	Serum (1.0 ml)	IgG+/IgM+	HEV RNA+/10^6^ copies/ml	3	14	J3
-	10% Phosphate-buffered saline (1.0 ml)	_	Undetectable	_	16	I2
					18	Q12

FALF^*^ – fatal acute liver failure; AH^**^ – acute hepatitis.

### Experimental design

Eight cynomolgus monkeys were intravenously inoculated with either Br-swine (monkeys X15, Q11, and I3), Dutch swine (O1, G3, and F3), Br-human (J3), or Ar-human (R7) viruses. As a control, 2 monkeys (Q12 and I2) were inoculated with a 10% phosphate-buffered saline (PBS) solution (pH 7.3). All of the animals had been previously screened for the anti-hepatitis A virus (HAV) and anti-HEV antibodies by the enzyme-linked immunosorbent assay (ELISA) using the Bioelisa HAV kit (Spain Biokit, Barcelona, Spain) and the IgG anti-HEV kit (MP Biomedicals, California, USA), respectively. During the study and quarantine periods, the monkeys were housed in an Animal Biosafety Level 2 facility. The animals were clinically monitored for 67 days post infection (dpi); they were monitored daily by the veterinary staff and checked weekly for rectal temperature and weight changes. Whole-blood samples (3 ml) were collected by venipuncture at 0, 7, 14, 25, 32, 39, 46, 53, and 67 dpi for HEV RNA and antibody detection. Faecal samples were collected at 0, 5, 7, 12, 14, 18, 21, 25, 27, 32, 35, 39, 42, 49, 53, 56, 63, and 67 dpi for HEV RNA detection and were stored in polystyrene tubes at -20°C until use. Salivary samples were collected from Q11, J3, O1, G3, F3, I3, and Q12 using OraSure^®^ collection devices (OraSure Technologies Incorporated, Pennsylvania, USA) at 0, 7, 14, 21, 28, 35, 42, and 53 dpi for IgA detection. For the collection of saliva and blood and liver biopsies (weekly), the animals were anaesthetised with ketamine hydrochloride at 20 mg/kg (Vetanarcol, König, Argentina) and xylazine hydrochloride at 0.1 mg/kg (Syntec Brazil, São Paulo, Brazil). At 67 dpi, all of the animals were euthanised under deep barbiturate anaesthesia with sodium thiopental 2.5% at 25 mg/kg (Thiopentax, Cristalia, São Paulo, Brazil), which was delivered intravenously. Subsequently, cardiac punctures were performed, and the animals were euthanised by exsanguination.

### ELISA tests

The presence of anti-HEV IgG was detected using a commercial ELISA Kit (MP Biomedicals, Ohio, USA) according to the manufacturer’s instructions. Pre- and post-inoculation samples were also tested for macaque anti-HEV IgG, IgM (sera), and IgA (saliva) using a modified protocol (Dr. Julio Moran Laboratories, Zurich, Switzerland) for the commercially available DiaCheck anti-human HEV antibody assay with an adapted goat anti-macaque immunoglobulin conjugate (Fitzgerald Industries International, Inc., Massachusetts, USA).

### Real-time PCR (qPCR)

Total RNA was extracted from 140 μl of a serum and faecal suspension (10% w/v in PBS) using the QIAamp Viral RNA Kit (Qiagen, California, USA). Reverse transcription was performed using 25 μl of RNA, 200 IU/ml Superscript III reverse transcriptase (Life technologies Corp., USA), and 20 pmol/μl random primer (Life technologies Corp., USA). A quantitative TaqMan real-time PCR assay was performed using a standard curve that was generated with a plasmid clone from a Brazilian swine strain that had been previously characterised as having genotype 3 [26]. Plasmid serial dilutions ranging from 10^8^ to 10^1^ were used to provide quantification parameters.

The qPCR reactions were performed in duplicate in a final volume of 25 μl consisting of 12.5 μl Universal PCR Master Mix (Life technologies Corp., USA), 5 μl cDNA, endonuclease-free water, and the previously described primers and probes [27]. The following conditions were used for the qPCR: 50°C for 2 min to activate the uracil N-glycosylase (UNG), an initial denaturation step at 95°C for 15 min, 45 cycles of denaturation at 95°C for 10 sec, and annealing/extension at 55°C for 1 min. The amplification data were collected and analysed with Applied Biosystems 7500 Software^®^ v2.0. Samples were considered to be positive when the signal crossed the threshold line, presenting a characteristic sigmoid curve. The number of viral genome copies (GC) was determined by adjusting the values according to the volumes that were used for each step of the procedure (i.e., extraction, cDNA synthesis, and the qPCR reaction).

### Liver function

Alanine aminotransferase (ALT), aspartate aminotransferase (AST), gamma-glutamyl transferase (GGT), and total bilirubin (Tb) serum levels were determined using a commercial enzymatic colorimetric method (Abbott, IL, USA). The baseline levels were obtained from blood samples of the pre-inoculation and control groups.

### Peripheral blood cell counts

Haematological, biochemical, and serological parameters were established for each animal using individual pre-inoculation samples. Blood samples were collected using ethylenediaminetetraacetic acid (EDTA) capillary tubes. Peripheral blood cells were counted using a commercial veterinary haematology analyser (Roche Diagnostica, Brazil).

### Histopathological analyses

Liver biopsies were collected at 0, 7, 14, 25, 32, 39, 46, 53, and 67 dpi by ultrasound-guided liver puncture, as described previously [28]. A portion of each sample was stored in 10% buffered formalin (pH 7.0) before being processed and embedded in paraffin according to standard methods. Paraffin blocks were sectioned at 4 μm and stained with haematoxylin-eosin, Giemsa stain, and Masson’s trichrome stain (Sigma-Aldrich, USA). The slides were examined under light microscopy, and inflammatory lesions were quantified using a scale from 0 to 4 that was based on the number of focal mononuclear cell infiltrates per 10 hepatic lobules: 0 = no inflammation, 1 = 1 to 2 focal infiltrates (poor), 2 = 3 to 5 focal infiltrates (mild), 3 = 6 to 10 focal infiltrates (moderate), and 4 = over 10 focal infiltrates (severe) as described previously [29]. A portion of each liver sample was stored in liquid nitrogen until the HEV analysis was performed. Pre-inoculation liver biopsies were performed to confirm the absence of other liver diseases.

### HEV antigen detection

Frozen liver sections (4 μm) that had been obtained at 67 dpi were examined by indirect immunofluorescence using a rabbit anti-HEV polyclonal antibody at a 1:150 dilution (Research Diagnostics Inc., USA) as the primary antibody (1 mg/ml), followed by a FITC-labelled goat anti-rabbit IgG antibody (2 mg/ml) at a 1:750 dilution (Sigma-Aldrich) as the secondary antibody. The slides were counterstained with Evans Blue, mounted with ProLong^©^ Gold Slow Fade in glycerol with DAPI stain (Life technologies Corp., USA), and covered with a coverslip. Images of the positive fields were obtained by confocal microscopy (LSM Zeiss 510 Meta Carl Zeiss, Oberkochen, Germany).

### Statistical analyses

Data are expressed as the means ± standard deviations (SD). Statistical analyses for the continuous variables were performed using 2-way ANOVA non-parametric statistics. A value of P < 0.05 was considered to be statistically significant. All of the calculations were performed using The R Project for Statistical Computing [30], and the graphics were produced using GraphPad InStat version 5.01 for Windows (GraphPad Software, San Diego, CA, USA).

## Results

### Cynomolgus monkeys inoculated with faecal suspensions from Brazilian and Dutch swine

#### ***Detection of HEV RNA in serum and faecal samples***

After inoculation with the Brazilian swine HEV (Br-swine), all of the animals were successfully infected. HEV RNA was detected by qPCR in the sera and faeces of all infected cynomolgus monkeys with the exception of monkey X15, which did not have viraemia but exhibited viral shedding in the faeces between 14 and 21 dpi. One animal showed a protracted pattern of viral shedding in the faeces from 21 dpi onwards. Out of the 3 animals that were inoculated with the Dutch swine HEV (D-swine), HEV RNA was detected at earlier time points in 2 (O1 and G3), with the virus being detected in the faeces at 5 or 7 dpi and in the sera at 7 to 14 dpi (Table 2).

**Table 2:** Summary of biochemical, histopathological, and virological patterns during HEV (genotype 3) infection in cynomolgus monkeys

**Inocula**	**Animal ID**	**ALT**	**AST**	**GGT**	**Tb**	**Liver injury	**HEV RNA detection**		***anti-HEV				Clinical interpretation****
		**(IU/dL) (**^**#**^**maximum value obtained)**				**(Score)**	(dpi*)		**(EIA reactivity)**				
									**Serum**			**Saliva**	
							**Serum**	**Faeces**	**IgM**	**IgG**	**IgA**	**IgA**	
Br-swine	X15	73	295	315	0.8	2	---	14 - 21	+	+	+	nt	Subclinical hepatitis E
	Q11	24	31	64	0.7	1	14	7 - 21	+	+	+	-	Subclinical hepatitis E without biochemical disorders
	I3	40	54	224	0.8	2	39 - 53	21 - 53	-	-	-	+	Subclinical hepatitis E
D-swine	01	40	119	237	0.8	1	14	5 - 18	+	+	+	-	Subclinical hepatitis E
	G3	32	42	138	0.8	1	7 - 14	7 - 18	+	+	+	+/-	Subclinical hepatitis E without biochemical disorders
	F3	99	302	238	0.7	1	---	---	+	+	+	+	Biochemical and histological hepatitis E
Br-human	J3	57/46	50	293	0.8	1	---	---	-	+	-	-	Biochemical and histological hepatitis E
Ar-human	R7	21	213	188	0.8	0	---	14 - 27	+	+	+	nt	Subclinical hepatitis E
Control	I2	21	43	92	0.8	0	---	---	-	-	-	nt	Uninfected
	Q12	21	116	103	0.7	0	---	---	-	-	-	-	Uninfected

*dpi: days post inoculation.

**Liver sections were prepared from large liver samples collected during necropsy; liver injury score (adapted from [29]): 0 – without inflammation, 1 – 1 to 2 lymphohistiocytic inflammatory infiltrates/10 liver lobules, 2–3 to 5 inflammatory infiltrates/10 lobules, 3–6 to 10 inflammatory infiltrates/10 lobules, 4- >10 inflammatory infiltrates/10 lobules (adapted from [29]).

***DiaCheck anti-human HEV antibody assay that was modified with a goat anti-macaque secondary antibody.

^#^The maximum value obtained during the entire follow-up period is provided.

****Subclinical hepatitis was considered when the animals presented only laboratorial finds of infection, Biochemical hepatitis was considered using species-specific normal values and pre-inoculation values. Liver enzymes values were elevated at least twice above baseline in the context of an HEV infection. Histological hepatitis was considered when the animals presented lymphohistiocytic inflammatory infiltrates in liver biopsy.

Br: Brazilian, Ar: Argentinian, D: Dutch, EIA: Enzyme immunoassay, nt: not tested, (they did not produced enough saliva).

### Biochemical and histopathological findings

Individual values for ALT, AST, GGT, and Tb did not vary significantly during the follow-up period. Exceptions included monkeys X15 (inoculated with the Br-swine virus) and F3 (inoculated with D-swine), of which serum levels for ALT, AST, and GGT were higher compared with their respective pre-inoculation values and the cynomolgus standard normal values (Table 2).

**Figure 1:**
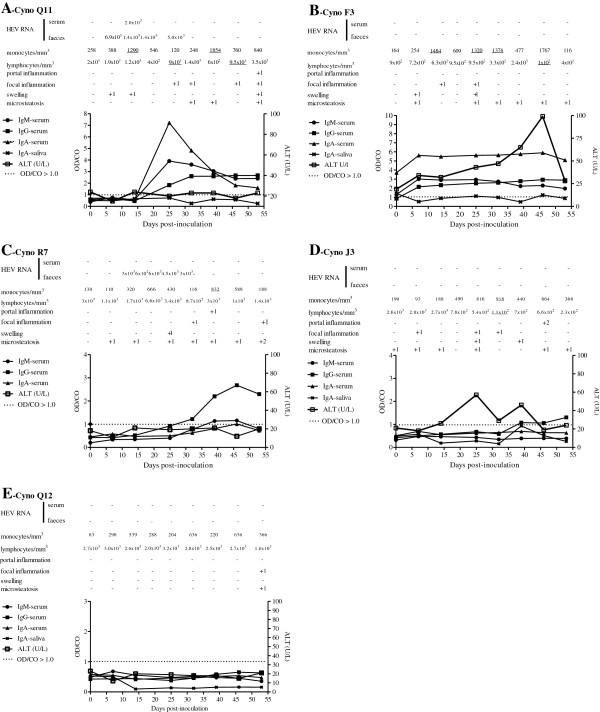
**Virological, inflammatory, biochemical, and antibody titre assessments from selected cynomolgus inoculated with HEV genotype 3.** The data for monkeys Q11, F3, R7, J3, and Q12 are represented in panels **A**, **B**, **C**, **D**, and **E**, respectively. The values of antibody levels are reported as optical density/cutoff ratios, with a OD/CO > 1.0 considered positive in the DiaCheck anti-human HEV antibody assay that was modified for use with goat anti-macaque secondary antibodies. Underlined values are considered changed compared with baseline levels.

The histological analyses of the pre-inoculation liver sections were normal in all animals. After inoculation, the most prominent inflammatory reaction was observed for monkey X15 (Brazilian inoculum), which exhibited a mild portal inflammation with lymphocytes and histiocytes. Monkeys I3 and Q11 showed mild to moderate hyperplasia of the Ito cells from day 0 throughout the follow-up period. Cynomolgus monkeys that were inoculated with the Dutch swine HEV showed changes that were compatible with biochemical and histological HEV-related hepatitis, including mild inflammatory reactions with multifocal lobular lymphohistiocytic infiltrates (Figure 1B and Table 2). Slight to mild fatty changes and mild diffuse swelling of the hepatic cells were detected in all of the animals, even in the absence of an inflammatory response. Monkeys I3 and Q11 showed mild to moderate Ito cell hyperplasia from day 0 until the end of the experiment (Figure 1A and Table 2).

### Detection of HEV antibodies in serum and saliva

**Figure 2:**
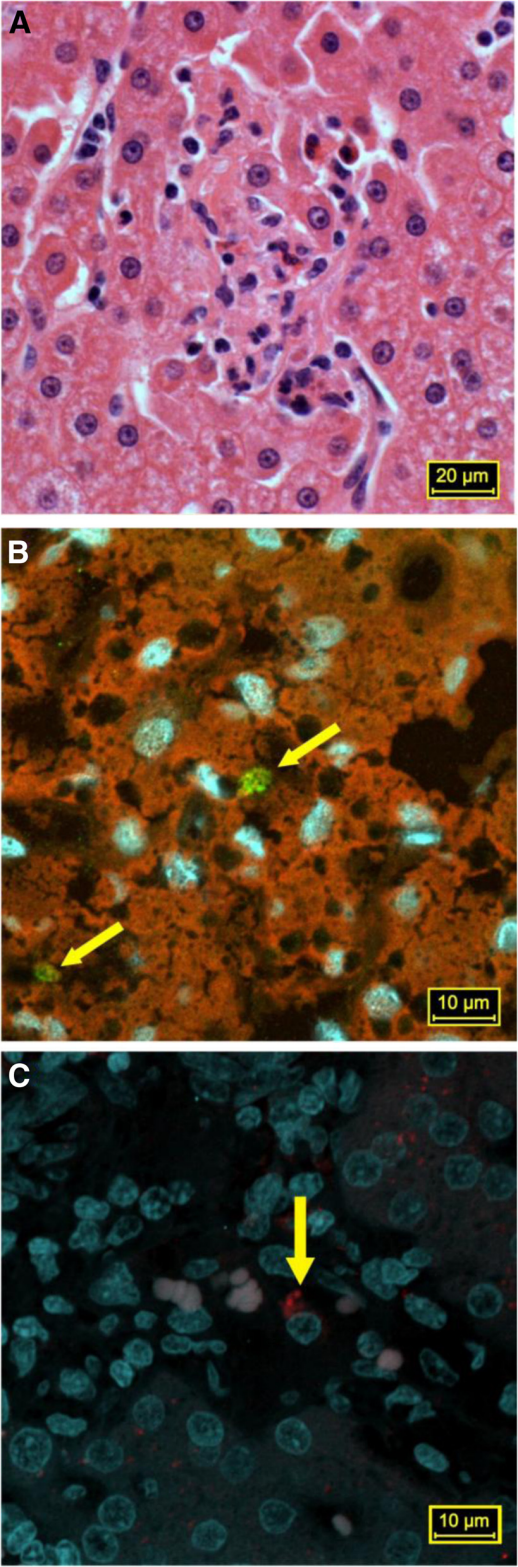
**Liver sections from cynomolgus (A and B) and Brazilian HEV infected pig (C). A**. Focal area of hepatocyte necrosis with polymorphic inflammation (histiocytes, lymphocytes, and neutrophils). Stained with hematoxylin and eosin; **B**. Detection of HEV antigen (marked in green) in the cytoplasm of hepatocytes and Kupffer cells at 67 dpi (cynomolgus G3); and **C**. Detection of HEV antigen (marked in red) in the cytoplasm of hepatocytes from a naturally infected pig obtained from a commercial pig farm in Brazil (inoculum).

Animals that were inoculated with the Br-swine inoculum (Figure 2C) exhibited specific antibody responses, with their IgG and IgM serum titres beginning to rise at 25 dpi. The exception was monkey I3, for which the only detectable antibody was salivary IgA.

All animals that were inoculated with the D-swine HEV showed IgM, IgG, and IgA anti-HEV titres in the serum samples that were collected during the follow-up period. Both IgA and IgM were transient and decreased after 46 and 55 dpi, respectively (Figure 1B). The IgG titre remained high until the end of the study. The highest salivary IgA titre was detected at 39 dpi. In monkey F3, the highest IgA (serum and saliva), IgM, and IgG titres were detected immediately following HEV inoculation. Focal necrosis (Figure 2A) and HEV Ag were detectable until 67 dpi (Figure 2B). Monkeys O1 and G3 seroconverted and had biochemical and histological hepatitis in association with transient viraemia and faecal shedding. In monkey F3, sudden rises in serum IgM, IgG, and IgA titres occurred at 7 dpi, suggesting a pre-exposure to HEV without detectable HEV antibodies in the pre-inoculation screening (Figure 1B).

### Cynomolgus monkeys inoculated with biological samples from patients from Brazil and Argentina

#### ***Cynomolgus monkey inoculated with Brazilian human HEV***

The animal that was inoculated with the Brazilian human virus (cynomolgus J3) exhibited rising levels of anti-HEV IgG antibodies in association with the development of inflammatory liver lesions, which was consistent with immunomediated liver injury (Table 2). Slight to mild fatty changes and mild diffuse swelling of the hepatic cells were found in the pre-inoculation liver sample in addition to those that were sampled throughout the experiment. At 46 and 53 dpi, this monkey showed mild portal infiltration, and at necropsy, the liver histopathology was scored as 1 using the severity assessment scoring system for inflammatory lesions (Table 2). Two slight variations in ALT levels were observed at 25 and 39 dpi. A change in GGT was detected at 25 dpi, and variations in TB were detected at 7, 14, and 32 dpi (Figure 1D).

#### ***Cynomolgus monkey inoculated with Argentinean human HEV***

Cynomolgus monkey R7 was inoculated with the Argentinean human HEV sample (Ar-human) and developed biochemical and histological hepatitis with viral shedding. Mild to moderate hyperplasia of the Ito cells and mild portal infiltration were observed at 25 dpi and 7 dpi, respectively. Liver samples that were obtained at necropsy showed histological recovery (Table 2 and Figure 1C).

### Negative controls

Both of the negative controls (monkeys Q12 and I2) developed non-significant biochemical alterations directly following the liver puncture procedures. Liver biopsies were scored as 0 using the severity assessment scoring system for inflammatory lesions (Figure 1E).

### Peripheral blood cell counts

**Figure 3:**
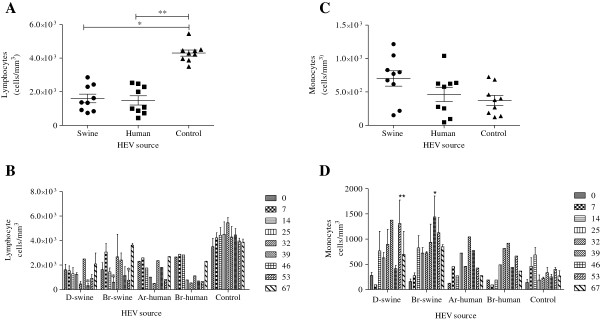
**Haematological changes in cynomolgus monkeys experimentally infected with HEV.** Lymphopenia was observed in the HEV-inoculated groups. An average lymphocyte was calculated at each of nine time points for all monkeys in each group **(A)** and in individuals **(B)**. A trend toward monocytosis was observed in the inoculated groups. An average monocyte count for all HEV-inoculated monkeys in each group **(C)**, and significant differences were observed in some individuals **(D)** (ANOVA, **P < 0.01, *P < 0.05).

In general, the average number of platelets and red blood cells did not change over the course of the experiment. However, severe episodes of lymphopenia (< 200 cells/mm^3^) were observed throughout the follow-up period. The average lymphocyte count was reduced (P < 0.01) in comparison with the baseline value of lymphocytes in the peripheral blood (2,800 ± 1,000 cells/ml) (Figures 3A and 3B). Discrete monocytosis (> 900 cells/mm^3^) was also observed in the inoculated animals (Figures 3C and 3D).

## Discussion

This article describes a successful, original approach for the experimental infection of cynomolgus macaques with HEV genotype 3 from different sources (human and swine) and different clinical manifestations. In their original hosts, these viruses were associated with varying clinical manifestations, ranging from none in the swine to fatal acute liver failure or acute hepatitis in the humans. HEV samples from different geographical regions, including Argentina, The Netherland and Brazil where hepatitis E is rare and/or under-diagnosed, were used in the present study. The intravenous route was employed for inoculation to ensure the best bioavailability of the inocula. Furthermore, our previous experience with the experimental transmission of enterically transmitted viral hepatitis in different non-human primates indicated that the only difference between oral and intravenous inoculation is the period of viral incubation, which is generally shorter for the intravenous route. Similar to the other experimental studies employing the cynomolgus monkey as a model for HEV infection, we found slight biochemical and histological alterations [31].

We observed that the first HEV-related event was viral shedding (at 5 to 21 dpi), as has been previously reported [32]. We assume that all of the animals presented with subclinical viral hepatitis independent of the source of the inoculum (swine or human) and the clinical presentation of the original host organism [22,31]. Even the animal that was inoculated with the Brazilian human HEV strain showed rising titres of anti-HEV IgG after 39 dpi. Indeed, there were no indications that the passive transfer of antibodies had occurred (Figure 1D). A late seroconversion to HEV (at 6–7 weeks pi) has previously been detected in cynomolgus monkeys that were inoculated intravenously with HEV from rabbits [33]. The absence of detectable HEV RNA in the faeces or sera may be explained by the presence of an IgG antibody response in the inoculum (human serum), which may have reduced the infectivity of the HEV particles, and/or by the lengthy time period that the samples had been stored. However, in both of these scenarios, the HEV peptides would have sensitised the macaque immune systems.

Cynomolgus monkey F3 presented with biochemical and histological hepatitis E (Table 2) without detectable viraemia or faecal shedding. However, early increases in antibody titres (IgM, IgA, and IgG) were observed concomitantly with slow and progressive ALT elevation. Apparently, the HEV infection was controlled in this monkey, accounting for the small number of focal inflammatory areas. A previous natural infection [34,35] or another HEV-like pathogen may explain this resistance to infection [36,37]. This result was intriguing because the main criterion for the inclusion of cynomolgus monkeys in our experimental protocol was the absence of anti-HEV IgG in the pre-inoculation serum. All of the animals that were provided by the animal breeding centre had been screened twice for HEV antibodies using two different assays. Sixteen out of the 76 captive cynomolgus monkeys were reactive to anti-HEV IgG in this screening and were therefore excluded from this study. All of the non-reactive animals were considered to be naive to HEV infection. Other authors have assumed that HEV or an HEV-related agent circulates naturally among Indian macaques [34,35,38,39]. A previous natural exposure may prevent a new (experimental) HEV infection [40,41]. In contrast, cynomolgus monkey I3 (Table 2) showed lymphopenia, prolonged viraemia (starting at 39 dpi), and faecal shedding (starting at 19 dpi) without seroconversion until the end of the experiment and possibly beyond that period. Such a pattern of prolonged viraemia and viral elimination without seroconversion or liver enzyme elevation has been described for HEV-infected, immunocompromised individuals [42-44], haemodialysis patients [45], and an immature neonate [44]. The protective roles of specific antibodies in controlling HEV infection have been confirmed in non-human primate studies [40,46-48] for both naturally acquired and experimentally induced infections [38,48,49] and in convalescent human patients [50]. The presence of activated lymphocytes during the resolution of hepatitis E infection has been demonstrated in acute hepatitis [51], acute liver failure [52], and acute hepatitis during pregnancy [53].

Interestingly, all of the infected monkeys showed severe lymphopenia and discrete monocytosis, coinciding with elevations in ALT and antibody titres. This study describes, for the first time, the haematological disorders that are associated with HEV infection, thus providing an additional parameter for future experimental protocols. In addition, we observed a progressive reduction in the average neutrophil counts of the inoculated animals compared with their baseline levels (data not shown). Other uncommon extra-hepatic manifestations, such as thrombocytopenia, anaemia [54], and neurological symptoms, have been previously described in HEV-infected patients [55]. Monocytosis has also been observed, typically in animals that have been inoculated with swine HEV. However, the restricted number of animals and inocula and the large individual variability among haematological values do not allow for precise conclusions to be drawn. The lymphohistiocytic hepatitis that was detected in our study has also been described in a rodent model of infection with HEV genotype 4 [56]. The elevated presence of infiltrated lymphocytes in the portal area and the focal inflammation that was detected in our study may be consequences of the intra-hepatic flow of lymphocytes, which was observed during this study for CD8+ cells in the cynomolgus monkeys with subclinical hepatitis E (data not shown). The same findings have also been reported in transplant patients with chronic hepatitis E [57] and in the liver parenchyma after HEV-induced acute liver failure [56]. Other authors have also observed lymphopenia in patients with hepatitis-virus-associated aplastic anaemia (HAA), suggesting that HAA may be influenced by the immune-mediated destruction of bone marrow [58]. Several viruses are considered to be triggers for autoimmunity and overt autoimmune disease. The virus-specific evasion mechanism, which is termed molecular mimicry, may serve as an accelerating factor for hepatic and extra-hepatic pathogenesis [59]. Further immunological approaches using peripheral blood cells and their precursors in bone marrow samples will contribute to understand the haematological manifestations of hepatitis E infection.

With the goal of improving HEV diagnoses, the serum titres for the IgA and IgM immunoglobulins were measured and showed similar profiles, with temporary increases being observed in most of the animals that were considered to be HEV-infected. Although the collection of salivary samples is less invasive than that of blood, the irregularities and fluctuations in the specific IgA levels in the saliva during the follow-up period indicate that this marker cannot replace classical testing that uses serum antibodies as markers for acute infection in routine diagnostics.

Our study indicate that HEV is capable of inter-species infection, which has been corroborated by other studies that have been conducted with HEV genotype 3 [21,60]. Because autochthonous hepatitis E cases have been associated with genotype 3 isolates, the zoonotic aspect of HEV infection should be considered in non-endemic, developing countries, such as Brazil and Argentina. Additional serological, molecular, and epidemiological studies will provide a better understanding of the dynamics and profile of this disease in Latin America.

## Conclusions

Finally, this work confirmed the ability of HEV to cross the species barrier for both the swine (Brazilian and Dutch) and human (Argentinean) strains, thus reinforcing the zoonotic risk of hepatitis E in South America. Cynomolgus monkeys that were infected with HEV genotype 3, obtained from different clinical presentations, developed subclinical hepatitis that was associated with haematological changes.

## Abbreviations

HEV: Hepatitis E virus; CEUA: Ethics committee for animal use; HAV: Hepatitis A virus; CONCEA: The national council for control of animal experimentation; SIV: Simian immunodeficiency virus; SRV/D: Simian type D retrovirus; HAA: Hepatitis-virus-associated aplastic anaemia; CECAL: Animal breeding centre; EDTA: Ethylenediaminetetraacetic acid; ALT: Alanine aminotransferase; AST: Aspartate aminotransferase; GGT: Gamma-glutamyl transferase; Tb: Total bilirubin.

## Competing interests

The authors declare that they have no competing interests.

## Authors’ contributions

MAP conceived the idea, managed the study, participated in the analysis of data and the preparation of the manuscript; LGC and DRLS participated in the study design and performed the experiments; WHMVP, JEG, and MSM provided the inocula; VSP and LGC performed the molecular biology tests; RSM and MP-M performed the histopathological and confocal analysis, respectively; LMP participated in the immunofluorescence analysis; JM: performed the serological tests; OGC realized the statistical analysis; MAP and JMO wrote the paper. All authors read and approved the final manuscript.

## Authors’ information

Please use the form below to submit correspondence to the authors or contact them at the following address.

Marcelo Alves Pinto, Laboratório de Desenvolvimento Tecnológico em Virologia, Instituto Oswaldo Cruz, Fundação Oswaldo Cruz, Ministério do Saúde, Governo Federal, Brasil. Pavilhão Helio e Peggy Pereira sala B221, Avenida Brasil 4365, Manguinhos, Rio de Janeiro, CEP 21045–900.

LM L, is fellowship of CNPq.

## Pre-publication history

The pre-publication history for this paper can be accessed here:

http://www.biomedcentral.com/1471-2334/13/495/prepub
